# Genetic links between multimorbidity and human aging

**DOI:** 10.1007/s11357-025-02044-3

**Published:** 2025-12-17

**Authors:** Phuong-Anh Dinh, HyeRim Han, Seungsoo Kim, Qinghua Guo, Zhengdong D. Zhang, Jan Vijg, Yousin Suh

**Affiliations:** 1 Department of Biological Sciences, Columbia University, New York, NY 10027 USA; 2 Department of Obstetrics and Gynecology, Columbia University Irving Medical Center, New York, NY 10032 USA; 3 Department of Genetics, Albert Einstein College of Medicine, New York, NY 10461 USA; 4 Department of Genetics and Development, Columbia University Irving Medical Center, New York, NY 10032 USA

**Keywords:** Aging, Age-related disease, Multimorbidity, Multivariate analysis, Geroscience

## Abstract

The growing epidemiological burden of multimorbidity among older adults underscores an urgent need to develop interventions that can address multiple age-related diseases (ARDs) at once. Yet, the biological mechanisms driving their co-occurrence remain poorly understood. In this study, we conducted a multivariate genome-wide association analysis to dissect the shared genetic architecture of five common ARDs: heart attack, high cholesterol, hypertension, stroke, and type 2 diabetes. We defined this shared genetic component as the multivariate age-related disease factor (mvARD) and identified 263 independent variants across 180 genomic loci associated with mvARD. These variants were significantly enriched for associations with extreme human longevity, lending empirical support for the geroscience hypothesis in humans. Integrative gene prioritization using transcriptome-wide association studies, colocalization analysis, and Mendelian randomization identified four high-confidence genes in blood—*DCAF16*, *PHF13*, *MGA*, and *GTF2B*—with putative causal roles on mvARD. Using two-sample Mendelian randomization, we also found several modifiable lifestyle factors, including body mass index and dietary intake, that causally influenced the risk for multiple ARDs. Together, our findings revealed a shared genetic basis for common ARDs that overlapped with the biology of human aging and pointed to potential molecular and behavioral targets for delaying disease onset and promoting healthy aging.

**Graphical Abstract:**

**Figure:**
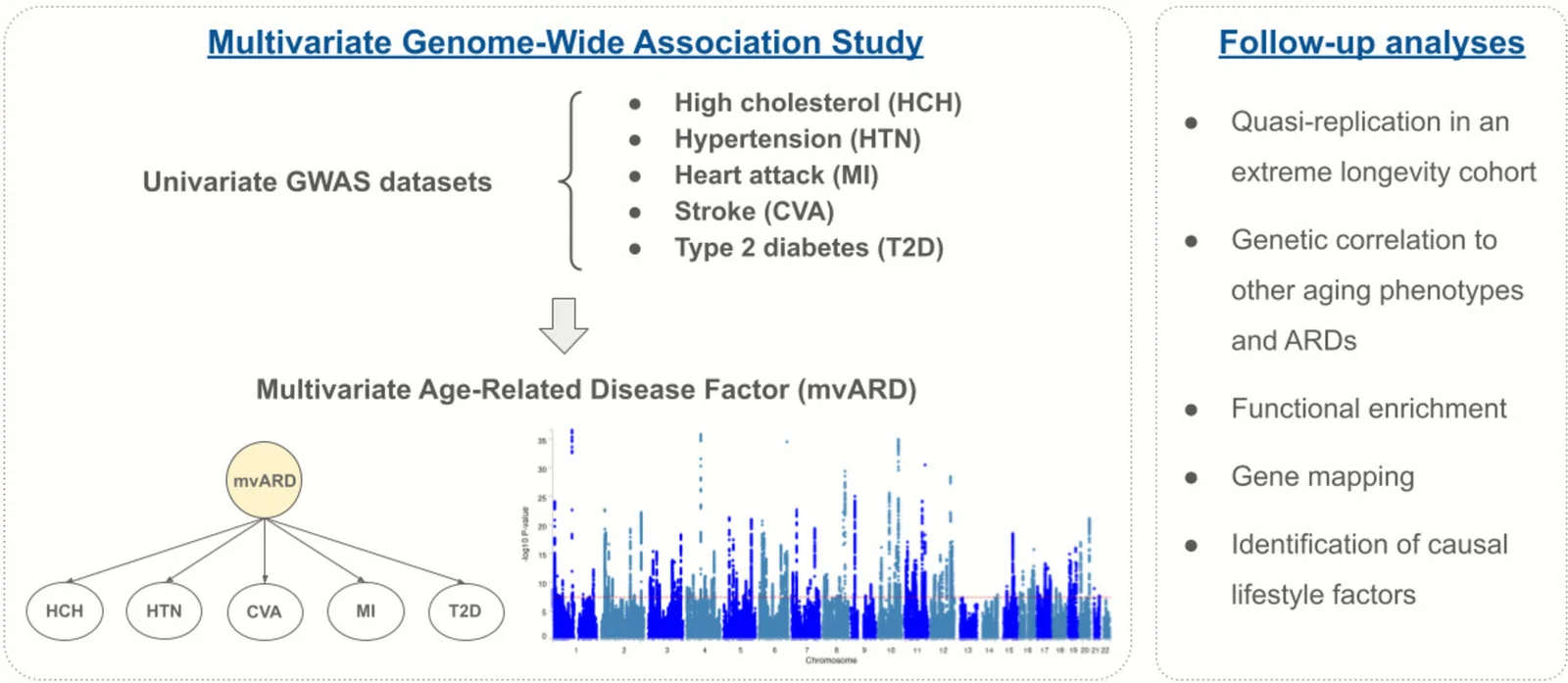

**Supplementary Information:**

The online version contains supplementary material available at https://doi.org/10.1007/s11357-025-02044-3.

## Introduction

As individuals age, their risk of developing multiple chronic diseases increases, resulting in a high prevalence of multimorbidity among older adults. In fact, more than 62% of those aged 65 and above are affected by at least two chronic conditions [1]. With the global population aging rapidly [2, 3], this burden is expected to intensify, posing a significant public health challenge. These trends underscore an urgent need to develop interventions that can address multiple conditions simultaneously. Yet, the biological mechanism that drives the co-occurrence of common age-related diseases (ARDs) remains poorly understood.

Traditionally, ARDs have been studied in isolation, with large-scale genome-wide association studies (GWAS) identifying hundreds of risk loci for individual conditions such as cardiovascular diseases [4–6], diabetes [7], and neurodegenerative disorders [8, 9]. While these studies have yielded valuable insights into disease-specific mechanisms, they fall short of capturing the shared genetic architecture that drives the frequent co-occurrence and genetic overlap of ARDs. To address this limitation, one promising strategy is genomic structural equation modeling (gSEM), a recently developed multivariate framework that integrates summary statistics from univariate GWAS to uncover the shared genetic liability across traits [10]. gSEM has been successfully applied in diverse domains, including externalizing behaviors [11], ageing phenotypes [12], pubertal timing [13], and alcohol consumption [14]. When applied to ARDs, this approach holds the potential to uncover the convergent biological mechanism that underlies age-related multimorbidity, thereby providing a foundation for developing interventions that can target multiple conditions at once.

In parallel, a longstanding body of research has focused on aging biology as the unifying driver of disease vulnerability and multimorbidity. This perspective, formalized as the geroscience hypothesis, proposes that since aging is the leading risk factor for multiple ARDs, targeting its biological drivers could delay or mitigate several conditions at once. Decades of studies in model organisms support this idea, demonstrating that genetic modulation of the fundamental biology of aging—often referred to as aging hallmark [15, 16]—can significantly extend both healthspan and lifespan. Elucidating the biological mechanism underlying human aging, however, has proven challenging. Due to limited effective sample sizes, only a small number of genetic loci have been identified across aging phenotypes, including extreme longevity [17–23], parental lifespan [24–28], frailty [29, 30], and healthspan [31]. This paucity of robust genetic associations has limited our opportunities for follow-up studies and mechanistic validation, leaving the geroscience hypothesis in humans a plausible yet largely untested model at the genetic level. By elucidating the shared genetic architecture of common ARDs, we gain a unique opportunity to empirically test this hypothesis. Specifically, we can assess whether variants associated with the shared genetic liability across multiple ARDs are also enriched for association with extreme longevity, but showing effects in the opposite direction. Evidence of such overlap would support a shared genetic basis between multimorbidity and aging, thereby providing genetic support for the geroscience hypothesis in humans.

While gSEM offers a promising avenue for genetic discovery, it also inherits the interpretative challenges common to GWAS. Increasing evidence shows that the vast majority (> 90%) of common variants associated with human traits lie in non-coding regions of the genome, where they are presumed to exert effects through regulatory mechanisms. Assigning function to these non-coding variants remains a major challenge, owing to limited knowledge of the regulatory elements involved, the genes they affect, and the specific cellular contexts in which they operate. In fact, difficulties in GWAS interpretation have been the major barrier to translating GWAS findings into mechanistic insights and therapeutic strategies.

**Fig. 1:**
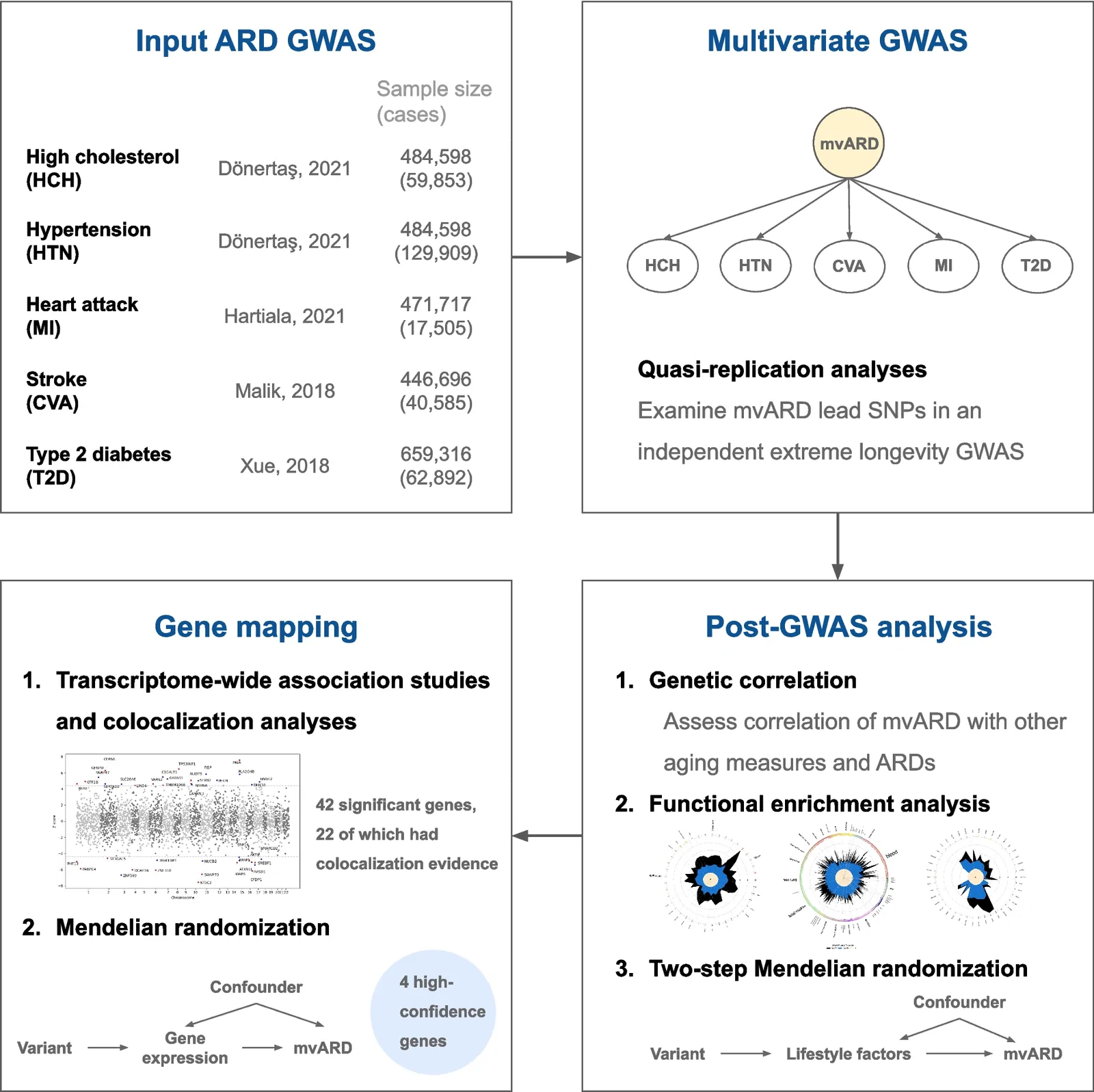
An overview of the study’s data source and analytical workflow

In this study, we applied gSEM to GWAS of common ARDs to identify genetic variants associated with their shared genetic architecture. To validate our findings and evaluate their relevance to aging, we conducted a quasi-replication analysis using an independent extreme longevity GWAS as a proxy phenotype. To explore the biological functions of the associated loci, we used GARFIELD [32] to assess their enrichment in various regulatory annotations and cell types. We then prioritized putative causal genes through three complementary approaches: transcriptome-wide association studies (TWAS), colocalization analysis, and Mendelian randomization (MR). Finally, we conducted two-sample MR to examine the causal effects of modifiable lifestyle factors on the shared genetic liability to ARDs. Together, these analyses highlighted potential biological pathways and behavioral targets for promoting healthy aging at the population level. An overview of our study design is provided in Fig. 1.

## Methods

### Selection of genetically correlated age-related diseases

To identify candidate ARDs for gSEM analysis, we used a published list of 25 common diseases whose age-of-onset profiles showed a sharp increase after age 40 [33]. To avoid redundancy between parent and child terms, we constructed a hierarchical graph and included only terminal diseases (Supplementary Fig. S1). For each disease, we manually selected summary statistics from either the GWAS Catalog or ref [33] based on the following criteria: 1) data from European population, 2) mean χ^2^ > 1.05, 3) h^2^ > 0.05, and 4) largest available sample size. If summary statistics were unavailable, we used the closest parent term. Broad disease categories, such as connective tissue disorders and renal/urology, were excluded to maintain specificity. This resulted in a final list of 11 diseases: atrial fibrillation [4], angina [33], cataract [34], diverticulitis [33], glaucoma [33], heart attack [6], high cholesterol [33], hypertension [33], osteoporosis [33], stroke [5], and type 2 diabetes [7]. All summary statistics were harmonized to GRCh37 and Ensembl release 113 using the gwas-sumstats-harmoniser tool prior to all analyses (https://github.com/EBISPOT/gwas-sumstats-harmoniser). To further refine the ARD list, we calculated the pairwise genetic correlations for the 11 included ARDs using LDSC [35] (Supplementary Table S1-2). We excluded diseases with weak genetic links (mean r_g_ < 0.2) and removed one from each highly correlated pair (r_g_ > 0.9) to avoid redundancy. This process resulted in a final list of five ARDs: heart attack (*N* = 471,717), high cholesterol (*N* = 484,598), hypertension (*N* = 484,598), stroke (*N* = 446,696), and type 2 diabetes (*N* = 659,316).

### Genomic SEM

gSEM is a multivariate framework that uses summary statistics to model the shared genetic architecture of complex traits. It is robust to sample overlap and sample size imbalance. The details of the method can be found in the original paper [10]. We used gSEM (v0.0.5) to estimate the genetic covariance matrix across 5 chosen ARDs and its sampling covariance. To guide the multivariate analysis, we performed exploratory factor analysis in R using the ‘factanal’ function with promax rotation, testing models with one and two latent factors. The one-factor model explained 39.6% of the variance while the two-factor model explained 55.9% of the variance. However, the second factor was primarily driven by hypertension (Supplementary Table S3). After iteratively removing loadings < 0.3, confirmatory factor analysis using genomic SEM showed that both models had similar fit (CFI = 0.97, SRMR = 0.04). We therefore selected the more parsimonious one-factor model (Supplementary Table S4). We then integrated individual autosomal SNPs into the genetic and sampling covariance matrix to estimate their effects and generate summary statistics for the common latent factor (mvARD). We followed recommendations from the gSEM package and restricted the summary statistics to variants with MAF between 10 and 40% to produce stable estimates of effect sample size. We then applied LDSC to estimate the SNP heritability and evaluate potential confounding of the generated mvARD summary statistics.

### Identification of genomic loci

We used FUMA [36] to identify independent genomic loci through double clumping of genome-wide significant variants (*P* < 5 × 10^–8^). Variants were initially clumped at r^2^ < 0.6 to define independent SNPs and set locus boundaries, then re-clumped at r^2^ < 0.1 to identify lead SNPs. Loci located within 250 kb of each other were subsequently merged. We considered a locus to be novel if its lead variants were located more than 1 Mb away from all genomic loci of contributing univariate GWAS.

### Quasi-replication analyses

We used the summary statistics from an extreme longevity GWAS where cases were defined as individuals who survived beyond the 90th percentile of their respective birth cohort [18] as a proxy phenotype to evaluate the validity of mvARD associations. We examined all 263 mvARD lead SNPs in this dataset through three complementary tests. First, we performed a sign discordance test to assess whether the direction of effect for each SNP was more frequently opposite than expected under the null hypothesis of random alignment (H_0_ = 0.5). For the second and third tests, we generated an empirical null distribution by matching each mvARD SNP to 250 independent SNPs (r^2^ < 0.1) of similar minor allele frequencies (± 1%). Using this null, we then (i) compared the proportion of nominally significant SNPs in the mvARD set versus the matched set via a binomial test, and (ii) assessed whether mvARD SNPs showed stronger overall association signals using a one-sided Mann–Whitney test. We strongly rejected the null hypotheses in all quasi-replication tests (Supplementary Table S9).

### Genetic correlation of mvARD to other aging measures and ARDs

To explore how mvARD relates to other aspects of aging, we collected summary statistics for three additional aging traits—healthspan [31], parental lifespan [24], and frailty index [29]—as well as 24 selected ARDs (Supplementary Table S10). Together with extreme longevity and the five input GWAS traits, we estimated their genetic correlations with mvARD using bivariate LDSC with default settings. A trait was considered significantly correlated with mvARD if it met the Bonferroni-corrected threshold of 1.5 × 10^–3^ (= 0.05/33).

### Functional enrichment analysis

To investigate the regulatory functions of mvARD-associated variants, we used GARFIELD [32] to test for enrichment across genomic annotations from the ENCODE and Roadmap Epigenomics projects. The analysis included DNase I hypersensitive sites, open chromatin peaks, transcription factor footprints, FAIRE, histone modifications, chromatin segmentation states, genic annotations, and transcription factor binding sites. Enrichment was assessed at two association *P*-value thresholds (*P* < 10^–8^ and *P* < 10^–5^) using default parameters.

### Transcriptome-wide association studies and colocalization analysis

To identify candidate target genes, we performed TWAS using FUSION [37]. We used precomputed gene expression prediction models derived from whole blood in the Young Finns Study, downloaded from the FUSION website. Genes with insufficient numbers of overlapping SNPs for reliable expression imputation were excluded, yielding 4,700 genes for analysis. A gene was considered significantly associated with mvARD if it passed the Bonferroni-corrected threshold of *P* < 1.06 × 10^–5^ (0.05/4700). We additionally performed colocalization analysis using the R package coloc, as implemented in FUSION, to identify genes with evidence of a shared causal variant between the mvARD and eQTL signals. To assess whether the significant TWAS genes had previously been linked to disease traits, we queried the GWAS Catalog and filtered associations based on the MAPPED_GENE and REPORTED GENE(S) fields.

### Mendelian randomization

#### Gene prioritization

To establish the causality of gene expression on mvARD, we performed MR using cis-eQTL from the eQTLGen consortium [38] as exposure variables. We selected independent IVs for each gene based on genome-wide significance (*P* < 5 × 10^–8^), low linkage disequilibrium (r2 < 0.01), and a minimum distance of 10 Mb between variants. We further excluded variants with F-statistics below 10 to reduce weak instrument bias. To estimate the MR effects, we applied multiple methods, including the Wald ratio (for genes with only 1 IV), the inverse variance weighted, weighted median, simple mode, and weighted mode. To test for horizontal pleiotropy, we applied the MR-Egger method and considered an intercept *P* value < 0.05 as statistically significant. We evaluate the heterogeneity across instruments’ estimates using Cochran’s Q test, with Q_pval < 0.05 indicating significant heterogeneity. To assess the direction of causality and filter out reverse causation, we applied the MR Steiger test. All analyses were conducted using the TwoSampleMR [39] package in R.

#### Modifiable lifestyle factors

We manually curated a list of modifiable lifestyle factors, grouped into 6 broad categories: body mass index (BMI), smoking, physical activities, sleep patterns, years of education, and dietary intake. For each factor, we selected one GWAS dataset from the IEU OpenGWAS database (Supplementary Table S14). We then identified independent IVs and applied MR using the same approaches as described in the gene prioritization section. A lifestyle factor was considered significantly associated with mvARD if any MR method passed the Bonferroni-corrected threshold of 2.4 × 10^–3^ (0.05/21), and nominally significant if *P* < 0.05.

### Analysis software

All software used in this study are publicly available without access restrictions. The R package GenomicSEM v0.0.5 is available at https://github.com/GenomicSEM/GenomicSEM. The LDSC package (v1.0.1) can be accessed at https://github.com/bulik/ldsc. The web-based platform FUMA is accessible at https://fuma.ctglab.nl/. The GARFIELD v2 source code is available at https://www.ebi.ac.uk/birney-srv/GARFIELD/. The TWAS FUSION software which includes coloc is available at http://gusevlab.org/projects/fusion/. Finally, the TwoSampleMR R package (v0.6.15) is accessible at https://mrcieu.github.io/TwoSampleMR/.

### Data availability

The sources of ARD GWAS summary statistics were listed in Supplementary Table S1. Additionally, summary statistics for extreme longevity, parental lifespan, frailty index, and healthspan were obtained from https://www.ebi.ac.uk/gwas/studies/GCST008598, https://www.ebi.ac.uk/gwas/studies/GCST009890, https://figshare.com/articles/dataset/Genome-Wide_Association_Study_of_the_Frailty_Index_-_Atkins_et_al_2019/9204998, and https://www.gwasarchive.org/, respectively. cis-eQTL summary statistics used for gene expression MR analysis were downloaded from the eQTLGen consortium (https://eqtlgen.org/phase1.html). Summary statistics used for lifestyle MR analysis were accessible via the IEU Open GWAS Project at https://gwas.mrcieu.ac.uk/ using the project ID listed in Supplementary Table S14. The summary statistic for the mvARD factor generated in this study is available at https://zenodo.org/records/15522815.

## Result

### Selecting genetically correlated age-related diseases

**Fig. 2:**
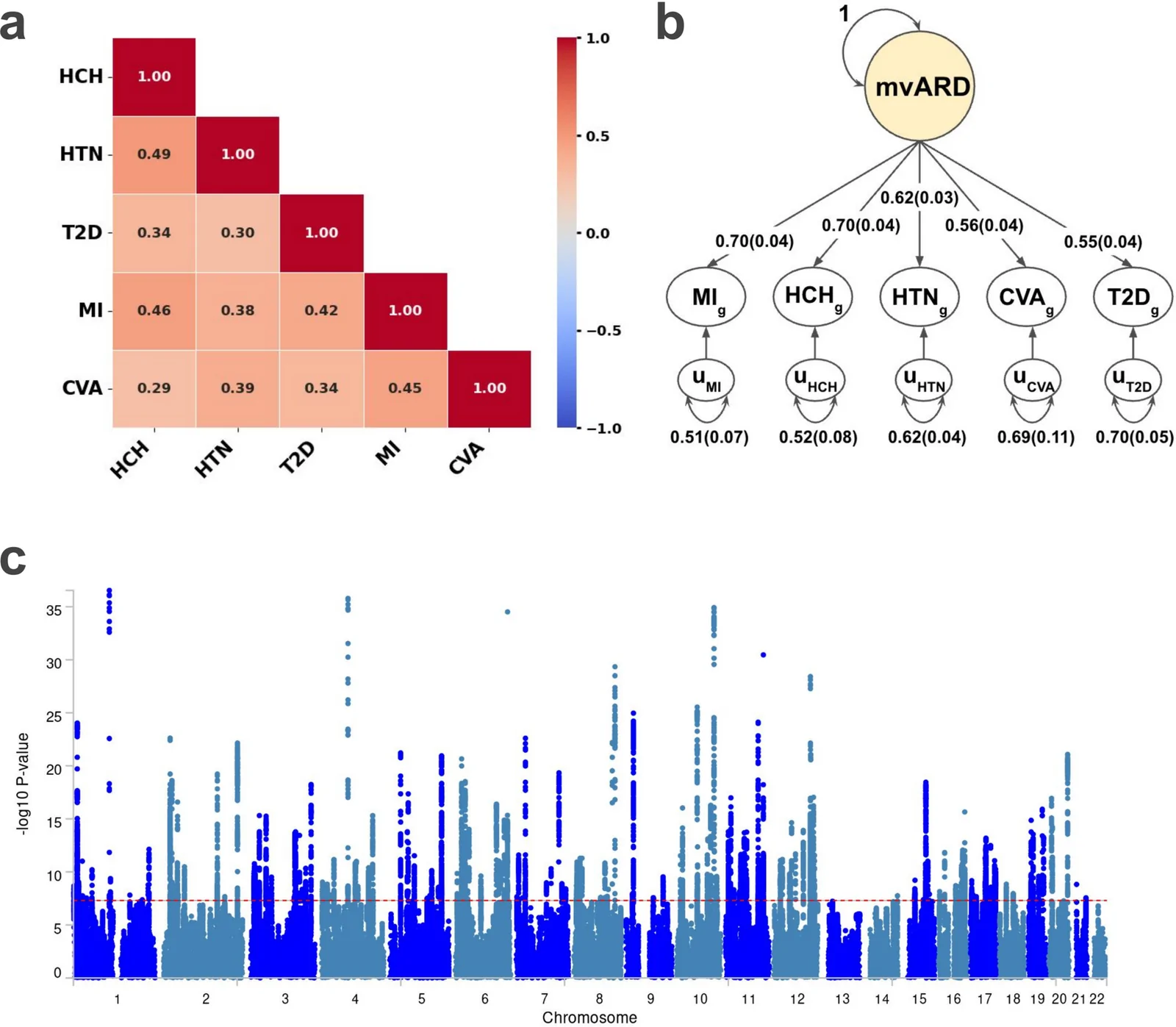
Multivariate analysis of age-related diseases. **a**. Pairwise genetic correlations between the 5 examined age-related diseases (ARD) were estimated using linkage disequilibrium score regression. **b**. The common factor model was estimated with gSEM and presented as a path diagram. Standardized factor loadings were shown with standard error in parentheses. **c**. Manhattan plot showing SNP associations (-log_10_(*p*-value)) with mvARD. The red dashed line represented the genome-wide significance level at 5 × 10^–8^. Abbreviations: MI—heart attack, HCH—high cholesterol, HTN—hypertension, CVA—stroke, T2D—type 2 diabetes

The elevating risk pattern of ARDs with advancing age may arise from two possibilities: a shared biological mechanism or distinct, time-dependent factors that independently affect each condition. To focus on ARDs that are likely to share common etiologies, we curated a subset of diseases that exhibit significant genetic correlations. More specifically, we included ARDs for which large-scale GWAS summary statistics were available and met our criteria for statistical power, heritability, and genetic correlation (Methods). This filtering process yielded a final list of 5 ARDs: heart attack (*N* = 471,717), high cholesterol (*N* = 484,598), hypertension (*N* = 484,598), stroke (*N* = 446,696), and type 2 diabetes (*N* = 659,316) (Supplementary Table S1). These conditions demonstrated strong positive genetic correlations with one another, indicating that susceptibility to one disease also predisposes individuals to the others (Fig. 2a, Supplementary Table S2).

### Modeling the shared genetic architecture of age-related diseases

To capture the shared genetic basis underlying the selected ARDs, we applied gSEM [10], a multivariate approach that models the genetic covariance structure across traits using GWAS summary statistics. We first performed exploratory factor analysis to identify the underlying factor structure, followed by confirmatory factor analysis, which confirmed that a common factor model provided a good fit to the data. The model demonstrated excellent fit indices with a comparative fit index (CFI) of 0.97 and a standardized root mean square residual (SRMR) of 0.04 (Fig. 2b, Supplementary Tables S3-4). This latent factor—termed mvARD (multivariate age-related disease factor)—captured 39.6% of the total genetic variance shared among the five ARDs. All diseases had significant and positive standardized factor loadings, indicating that they each contributed to the shared genetic signal and supporting the existence of a unified genetic basis for multiple ARDs.

### Multivariate GWAS

We conducted a multivariate GWAS analysis to estimate the association between 4,893,454 SNPs and the mvARD factor. The mean ꭓ^2^ and λ_GC_ of the resulting summary statistics were 1.94 and 1.58, respectively. The intercept estimated by LD score regression was 0.99 (se = 0.02), suggesting that the inflated test statistics were due to polygenicity rather than confounding factors such as population stratification [40]. The heritability of common SNPs on the observed scale was 9.17% (se = 0.37%). The estimated effective sample size for the mvARD factor was 475,203 where variants were constrained to have minor allele frequencies between 10 and 40% to produce a more stable estimate [10]. At the conventional genome-wide significance threshold (*P* < 5 × 10^–8^), we identified 263 lead SNPs across 180 genomic loci (independent R^2^ < 0.1 and distance ≥ 250 KB) (Fig. 2c, Supplementary Table S5-6). Many of these lead variants have been previously implicated in a broad range of diseases in the GWAS Catalog, with some linked to hundreds of known conditions (Supplementary Table S8). Among the 180 loci, six had not been reported in any of the five input GWAS. Of these, four variants (rs1788785, rs10931284, rs6505080, rs72887634) had known associations with several traits in the GWAS catalog, including lung function, brain morphology, and blood composition (Supplementary Table S6). One variant (rs16959812) had no known association, while the other (rs9430037) was linked to educational attainment.

We evaluated the Q_SNP_ statistics within the gSEM framework to determine whether each SNP influenced all five ARDs exclusively via mvARD or through independent pathways. The Q_SNP_ test follows a ꭓ^2^ distribution, and a significant Q_SNP_ value suggests that the SNP’s effect is likely not mediated by a common mechanism but rather disease-specific pathways. Out of the 263 lead SNPs, 98 (37%) showed significant heterogeneity (*P* < 0.05/263), suggesting they may influence ARDs via distinct pathways (Supplementary Table S5). To focus our downstream functional analyses on variants most representative of the shared genetic basis, we conservatively excluded all SNPs with nominal Q_SNP_ significance, resulting in a final set of 4,377,831 variants.

### Quasi-replication analyses

We cannot directly replicate findings from the mvARD factor as there is no equivalent phenotype. However, we expected that SNPs which increased the risk for multiple common ARDs would decrease the likelihood of being long-lived and vice versa. This expectation is grounded in the idea that mvARD captures a latent genetic liability related to systemic aging, as it aggregates genetic risk shared across prevalent ARDs. We therefore used the summary statistics from an independent extreme longevity cohort (*N* = 36,745) [18] as a proxy phenotype [41] for our quasi-replication analysis [42] (Supplementary Table S9). First, we assessed whether the 263 lead SNPs showed the expected sign discordance—that is, whether SNPs associated with higher mvARD corresponded to lower odds of extreme longevity, and vice versa. Consistent with our hypothesis, 76% of SNPs showed the expected direction of effect (P_one-sided_ = 1.36 × 10^–17^). To conduct the second and third analyses, we generated an empirical null distribution by randomly sampling 250 independent SNPs (r^2^ < 0.1), matched on minor allele frequency, for each of the 263 SNPs. In the second test, we found that a higher proportion of mvARD lead SNPs were nominally associated with extreme longevity compared to the null SNPs (18.6% vs 8.4%, P_one-sided_ = 1.17 × 10^–7^). In the third test, the 263 SNPs showed significantly stronger association signals than expected under the null (Mann–Whitney P_one-sided_ = 9.73 × 10^–12^). When taken together, these findings support the conclusion that mvARD associations are not spurious overall and are indeed enriched for genetic signals associated with extreme longevity.

### Genetic correlation to other aging measures and ARDs

To further investigate the relationship between the mvARD factor and aging, we estimated its genetic correlation with three different aging phenotypes: parental lifespan, healthspan, and frailty index. The mvARD factor showed strong genetic correlations with all three traits (parental lifespan: r_g_ = −0.65, *p* = 6 × 10^–122^; healthspan: r_g_ = 0.65, *p* = 9 × 10^–79^; frailty index: r_g_ = 0.60, *p* = 8 × 10^–116^). As expected, mvARD was also highly correlated with all five input GWAS traits (r_g_ = 0.79—0.95). In addition, we examined its correlation with other ARDs and found significant but weaker correlations with several conditions, including osteoarthritis, emphysema, and gout (Supplementary Table S10). It is worth noting that the absence of significant genome-wide correlations for some ARDs does not preclude a biological link, as opposing effects at unrelated loci may obscure associations driven by aging-related mechanisms [43].

### Functional enrichment analysis

**Fig. 3:**
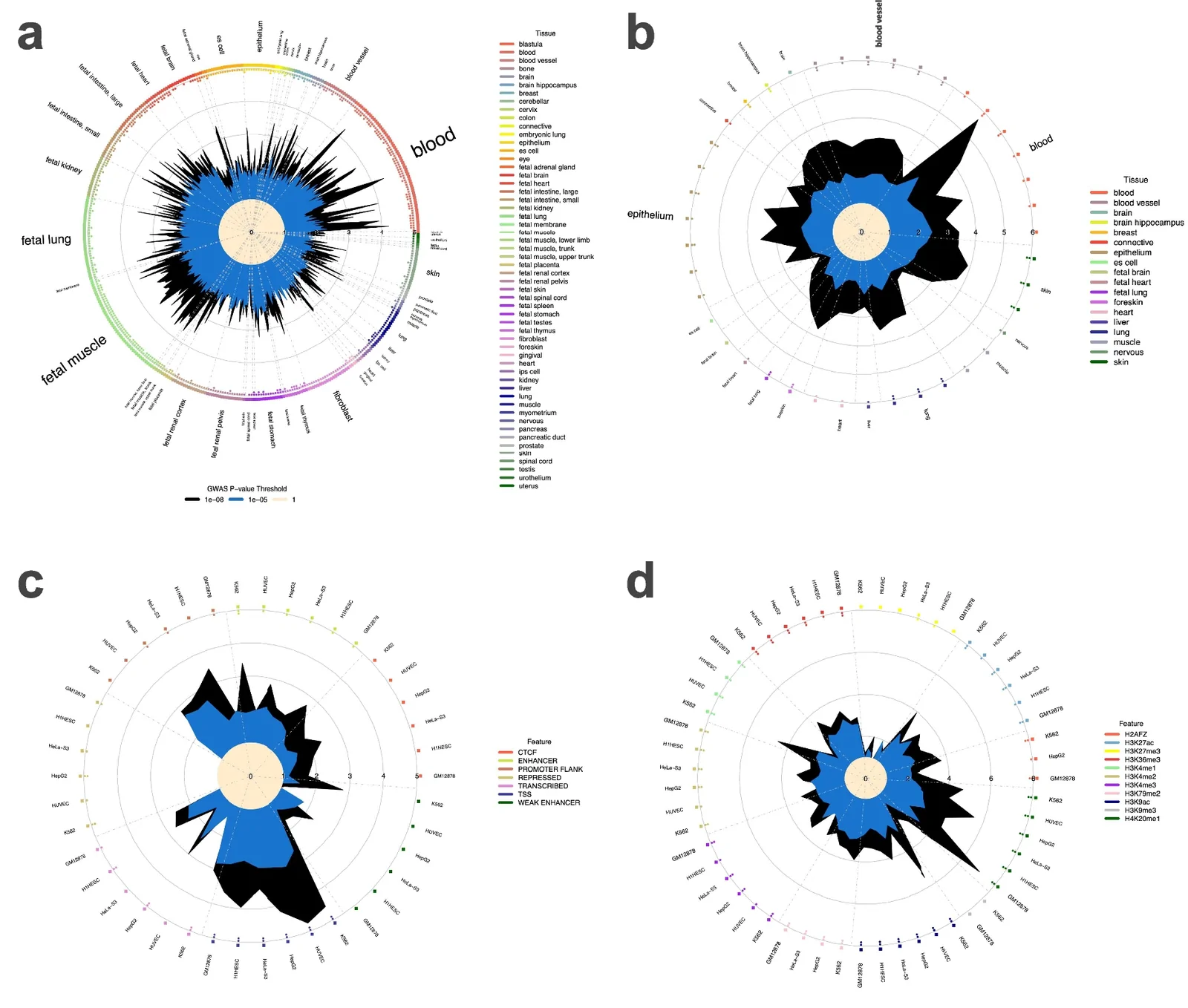
Functional enrichment analysis of mvARD. Enrichment of mvARD GWAS signals in (**a**) DNase I Hypersensitive Site (DHS), (**b**) transcription factor footprint, (**c**) chromatin segmentation state, and (**d**) histone modifications across all ENCODE and Epigenomic DHS cell lines. Radial lines represent the significance of odds ratios (OR) values at different *P*-value thresholds. Dots on the inner ring of the circular plot indicate annotations with significant enrichment after multiple testing correction, with the innermost ring corresponding to *P* < 10^–8^ and the outermost ring to *P* < 10^–5^

Over 90% of candidate SNPs for the mvARD factor were located in non-coding regions of the genome, consistent with observations from previous GWAS (Supplementary Fig. S2). To investigate the potential regulatory roles of these variants, we performed a functional enrichment analysis using GARFIELD, which takes into account linkage disequilibrium and gene density to reduce false-positive discovery (Supplementary Table S11). This approach evaluated whether mvARD-associated SNPs were overrepresented in specific regulatory and functional annotations across diverse cell types profiled by the ENCODE and Roadmap Epigenomics projects. We observed an overall enrichment of chromatin accessibility peaks and DNase I hypersensitive sites (DHS) in multiple tissues, suggesting that mvARD variants may exert their regulatory effects across diverse biological contexts, potentially reflecting the systemic nature of ARD mechanisms (Fig. 3a, Supplementary Fig. S3). Among all tissues, blood-derived cell types exhibited consistently strong enrichment signals, supported by multiple lines of evidence including DHS hotspots, open chromatin peaks, formaldehyde-assisted isolation of regulatory elements, and transcription factor footprinting (Fig. 3a-b, Supplementary Fig. S3-4). We also detected significant enrichment in chromatin states associated with active regulatory elements, particularly transcription start sites (TSS) and enhancers (Fig. 3c), as well as in regions marked by key histone modifications involved in transcriptional regulation, including H4K20me1, H3K9ac, and H3K4me1 (Fig. 3d). Together, these results suggest that mvARD variants are likely to exert their effects through gene regulation and transcription control. We decided to focus on blood in subsequent analyses due to its consistent enrichment across multiple annotations and its potential to exert systemic effects on multiple tissues.

### Transcriptome-wide association studies and colocalization analyses

**Fig. 4:**
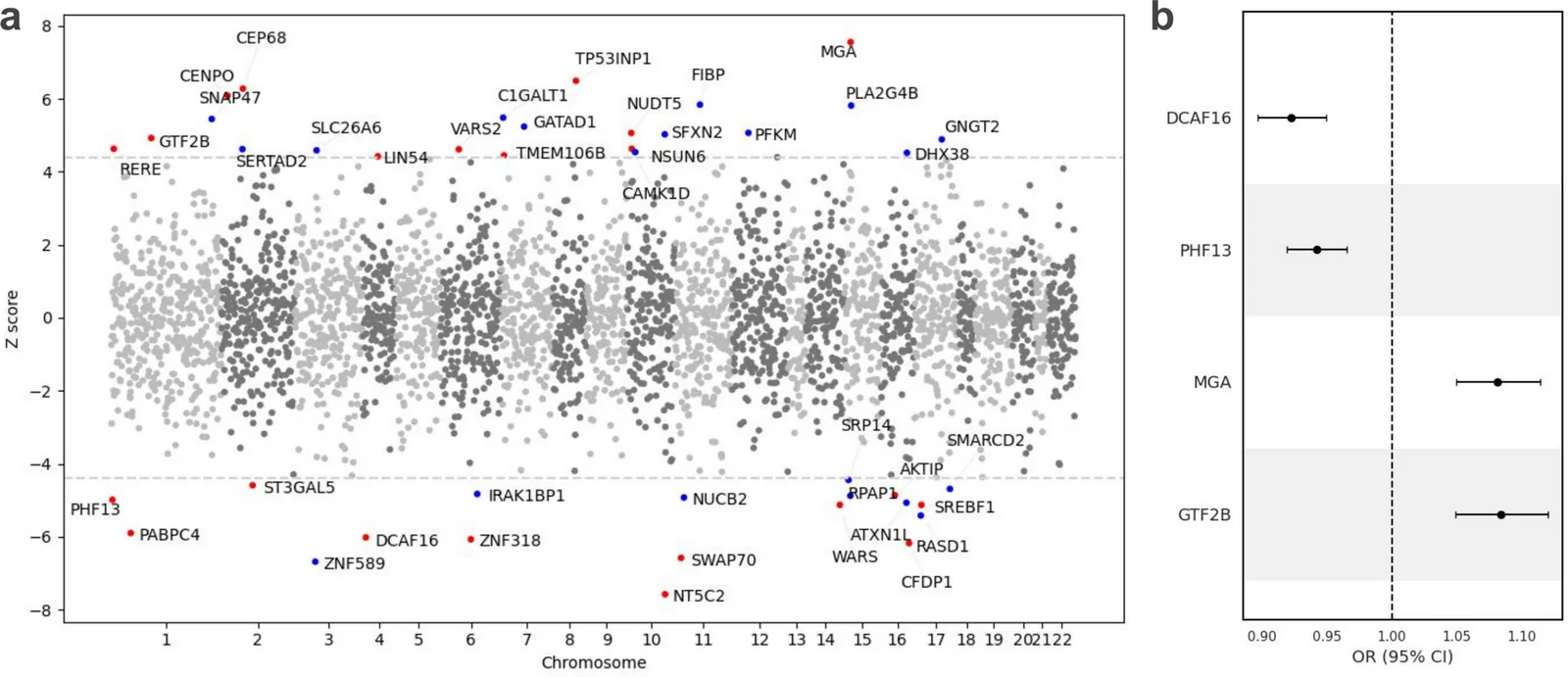
Transcriptome-wide association studies and Mendelian randomization. **a.** Manhattan plot showing gene associations (Z-score) with mvARD. The horizontal dashed line represents the Bonferroni-corrected significance threshold at *P* < 1.06 × 10^–5^. Genes surpassing this threshold are shown in blue and annotated with gene names. Among them, genes shown in red have additional support from colocalization analysis (PP4 > 0.8). **b**. Forest plot showing odds ratios (ORs) with 95% confidence intervals from Mendelian randomization for four high-confidence genes supported by TWAS, colocalization, and MR. The dashed vertical line indicates the null effect (OR = 1)

To identify candidate genes whose expression levels are associated with the mvARD factor, we conducted TWAS using FUSION [37], integrating mvARD GWAS summary statistics with gene expression prediction models derived from whole blood in the Young Finns cohort. After Bonferroni correction, we identified 42 genes whose expressions were significantly associated with mvARD (Fig. 4a, Supplementary Table S12). To further support these findings, we performed colocalization analyses [44] to assess whether mvARD and each gene’s eQTL share a common causal variant. We found strong colocalization evidence for 22 out of the 42 genes with a posterior probability (PP4) greater than 0.8, indicating a high likelihood that mvARD and gene expression are influenced by the same variant. Many of these 22 genes have previously been implicated in aging-related processes and pathways. For example, *AKTIP* is involved in telomere maintenance [45], *NT5C2* in AMPK signaling [46], *PHF13* in DNA damage response [47], and *SREBF1* in cellular senescence [48].

### Mendelian randomization

To complement TWAS and further evaluate the causal relationship between gene expression and mvARD, we performed MR analyses using eQTLs as exposure variables. We obtained valid instrumental variables (IV) for 10,837 genes using summary statistics from the eQTLGen consortium (Methods). At the Bonferroni-corrected significance level (4.6 × 10^–6^), we identified 67 genes whose expression levels show evidence of having causal effects on mvARD (Supplementary Table S13). Among these genes, four were also significant in the TWAS analysis and showed strong colocalization evidence (PP4 > 0.8). These four genes—*DCAF16*, *PHF13*, *MGA*, and *GTF2B*—thus represented high-confidence candidates with converging support from TWAS association, colocalization, and causal inference analyses (Fig. 4b).

### Modifiable dietary factors

Identifying causal links between lifestyle behaviors and mvARD is critical from a public health perspective, as such insights can guide strategies to promote healthy aging at the population level. With this motivation in mind, we applied MR to evaluate the potential causal effects of modifiable factors—including body mass index (BMI), smoking, physical activity, sleep patterns, years of education, and dietary intake—on mvARD (Supplementary Table S14). At the Bonferroni-corrected significance threshold, we found that higher BMI, coffee intake, and tea intake causally increased mvARD, indicating a greater risk for multiple ARDs. In contrast, more years of education showed a protective effect. We also identified several nominally significant (*P* < 0.05) factors that increase mvARD, including smoking initiation, insomnia, and red meat consumption.

## Discussion

In this study, we applied a geroscience approach to dissect the shared genetic mechanisms across common ARDs. We focused on a subset of genetically correlated conditions and showed that their common genetic risk can be captured by a single factor, which we termed mvARD. We acknowledged that the final set of ARDs included in our analysis consisted primarily of cardiometabolic conditions, which raised concerns about the generalizability of mvARD to a broader spectrum of ARDs. This selection reflected a methodological constraint of the gSEM framework, which requires significant global genetic correlations among traits [10]. As a result, diseases with weaker genetic links to cardiometabolic traits were excluded. It is important to emphasize that the exclusion of certain diseases from the gSEM analysis does not imply that they are unrelated to aging, as opposing effect directions at loci unrelated to aging can obscure genome-wide signals driven by aging-related mechanisms [43, 49]. Thus, the lack of detectable global correlation between some conditions and our selected ARDs may reflect technical limitations rather than true biological independence. Despite being derived from only five ARDs, mvARD demonstrated strong genetic correlations with multiple aging phenotypes, including healthspan, parental lifespan, and frailty index, supporting its broader relevance to human aging. In addition, mvARD showed moderate but significant genetic correlations with several other ARDs not included in our model, such as osteoarthritis, emphysema, and gout, further highlighting its potential to capture systemic aging biology beyond cardiometabolic risk.

To identify variants associated with mvARD and, therefore, multiple ARDs, we conducted a multivariate GWAS and identified 263 lead variants. Test statistics (ꭓ^2^, λ_GC_, and LDSC intercept) indicated that the analysis was well-powered with minimal evidence of confounding due to population stratification. To our knowledge, this is the first time a multivariate gSEM approach has been used to uncover the shared genetic basis of age-related multimorbidity. Notably, our approach uncovered substantially more genome-wide significant loci than any univariate aging GWAS, likely reflecting the enhanced statistical power gained by aggregating data from multiple large-scale ARD GWAS.

To demonstrate that our results were not spurious, we carried out a quasi-replication analysis using extreme human longevity as a proxy phenotype. We found that mvARD-associated variants were significantly enriched for longevity-linked signals, often with effects in the opposite direction. This means that individuals who have a lower risk for multiple ARDs are more likely to live longer, and vice versa, suggesting a shared genetic basis between multimorbidity and extended lifespan. Since longevity serves as a proxy for slower biological aging, our findings imply that decelerating the aging process could help prevent or delay the onset of multiple conditions. Our results also offer preliminary evidence that the shared genetic risks across ARDs converge toward a common genetic signature that reflects the pace of biological aging. Together, our findings extend prior observations of negative genetic correlations between individual ARDs and longevity, and provide empirical support for the geroscience hypothesis in humans.

Next, we performed a functional enrichment analysis using GARFIELD and observed significant enrichment of mvARD-associated variants in DHS across multiple tissues, supporting the view that mvARD captures systemic rather than tissue-specific aging. While we prioritized blood for variant-to-gene mapping due to its consistent and strong enrichment, future analyses could potentially incorporate tissue-specific mapping to uncover additional context-dependent effects. To identify candidate genes mediating the genetic risk shared across multiple ARDs, we applied three complementary approaches: TWAS, colocalization analysis, and MR. We used two independent eQTL reference panels to increase the robustness and reproducibility of our gene-level inferences: the Young Finns cohort for TWAS and colocalization analyses, and the larger eQTLGen consortium for Mendelian randomization. Four genes—*DCAF16*, *PHF13*, *MGA*, and *GTF2B*—emerged with convergent support across all three analyses, suggesting they are strong candidates mediating the risk of age-related multimorbidity.

Given their predicted roles in mediating the genetic risks across multiple ARDs, we expected that these genes would influence the fundamental aging processes. Indeed, three of the four genes have been previously implicated in biological pathways that align with key hallmarks of aging. *DCAF16* encodes a substrate recognition component of the CUL4-DDB1 E3 ubiquitin ligase complex, which mediates the degradation of nuclear proteins [50] and thus may contribute to the regulation of protein homeostasis. *MGA* is a transcription factor that represses MYC-target genes, and its conditional knockout in mouse hematopoietic cells has been shown to increase the expression of MYC and E2F targets, cell cycle genes, and mTOR signaling while downregulating TGFβ and other extracellular signaling pathways [51]. *PHF13*, also known as *SPOC1*, plays a key role in the DNA damage response [47] and helps regulate chromatin organization through its transcriptional co-regulatory function [52]. Finally, *GTF2B* is a general transcription factor involved in RNA polymerase II-mediated transcription initiation, and its role in ARDs has been less explored, likely due to its ubiquitous expression and essential function in core transcriptional processes. Together, these findings highlight promising gene-level targets for future mechanistic studies.

To experimentally validate the predicted causal relationships and establish the direction of effect for each gene, future studies could employ a multi-tiered functional genomics approach using targeted perturbations. Given the absence of a murine ortholog for *DCAF16* [50], its role could be initially investigated using in vitro models such as hematopoietic K562 or vascular-derived HUVEC cells. Systematic modulation of expression followed by multi-omics profiling could help identify downstream pathways, with a particular focus on aging hallmarks, including cellular senescence, loss of proteostasis, and genomic instability [15, 16], among others. These molecular readouts could then be complemented by phenotypic assays assessing DNA damage response, stress resistance, and cellular senescence to confirm functional impact.

Similarly, in vitro perturbations of *MGA*, *PHF13*, and *GTF2B* could empirically determine their directional effects on cellular aging. This is especially pertinent for *MGA*, given its opposing effects on distinct aging-related pathways. The specific mode of gene modulation—whether knockdown or overexpression—that consistently reduces the rate of senescence-associated or age-related cellular phenotypes without inducing toxicity could then be prioritized for in vivo validation in mouse models. Ultimately, lifespan and healthspan assessments, combined with tissue-specific molecular profiling, would clarify how these genes influence systemic aging and multimorbidity [53–55].

To inform potential public health strategies and clinical interventions, we investigated modifiable lifestyle and dietary factors and identified several exposures with evidence of causal relevance to mvARD. We found that higher BMI, coffee intake, and tea intake were associated with increased genetic risk for multiple ARDs, whereas more years of education had a protective effect. We also observed nominal associations for insomnia, smoking initiation, and red meat consumption. These results are generally consistent with prior epidemiological studies [56–64] linking these factors to multimorbidity, healthspan, and longevity, thus highlighting potential intervention targets for reducing ARDs burden at the population level.

Our study has several limitations. First, mvARD is a latent construct that represents the broad genetic liabilities to multiple ARDs and thus lacks the conventional units [10]. This complicates our interpretation of the effect size from MR analyses, particularly in a clinical context. Second, gSEM assumes an additive genetic model, which may overlook effects from recessive or more complex genetic patterns [10]. Third, our analysis is restricted to individuals of European ancestry, which limits the generalizability of our findings to other populations, as allelic frequencies, linkage disequilibrium structures, and genetic architectures can differ substantially across populations [65]. Fourth, our colocalization analysis assumes a single causal variant per locus, which may not always hold true. Fifth, due to the limited number of IVs for certain exposures, we are unable to conduct sensitivity analyses such as MR-Egger and Cochran’s Q. Sixth, our MR analyses of lifestyle factors assume linear exposure-outcome relationships [66], which may not capture the true underlying biology in many cases. For example, both coffee and tea consumption have been reported to exhibit nonlinear associations with all-cause mortality [67]. Finally, due to data availability, some of the lifestyle GWAS used in our analyses are based on UK Biobank data. Since the univariate GWAS underlying mvARD also draws from the same cohort, this sample overlap could potentially bias our MR estimates [68].

In conclusion, we applied a multivariate approach to dissect the shared genetic architecture of common ARDs and found strong support for the geroscience hypothesis in humans. Our integrative analyses identified candidate genes with potential causal roles in multimorbidity, offering targets for future mechanistic studies. In parallel, the identification of modifiable lifestyle factors points to actionable opportunities for disease prevention and health promotion at the population level.

## Supplementary Information

Below is the link to the electronic supplementary material.

ESM 1 (PDF 1.06 MB)

ESM 2 (XLSX 3.94 MB)
