# Supplementary material for: Genetic links between multimorbidity and human aging
Source: GeroScience. 2025 Dec 17;48(5):7277–92. doi: 10.1007/s11357-025-02044-3 (PMC13601487; doi:10.1007/s11357-025-02044-3)
Supplement: Supplementary file 1 [file 11357_2025_2044_MOESM1_ESM.pdf]

Article Title: Genetic links between multimorbidity and human aging

Journal Name: GeroScience

Author Names: Phuong-Anh Dinh[1], HyeRim Han[2], Seungsoo Kim[2], Qinghua Guo[2], Zhengdong D. Zhang[3], Jan Vijg[3], Yousin Suh[2,4,*]

Affiliations

[1] Department of Biological Sciences, Columbia University, NY 10027, USA

[2] Department of Obstetrics and Gynecology, Columbia University Irving Medical Center, New York, NY 10032, USA

[3] Department of Genetics, Albert Einstein College of Medicine, New York, NY 10461, USA

[4] Department of Genetics and Development, Columbia University Irving Medical Center, New York, NY 10032, USA

Corresponding author: Yousin Suh (ys3214@cumc.columbia.edu)

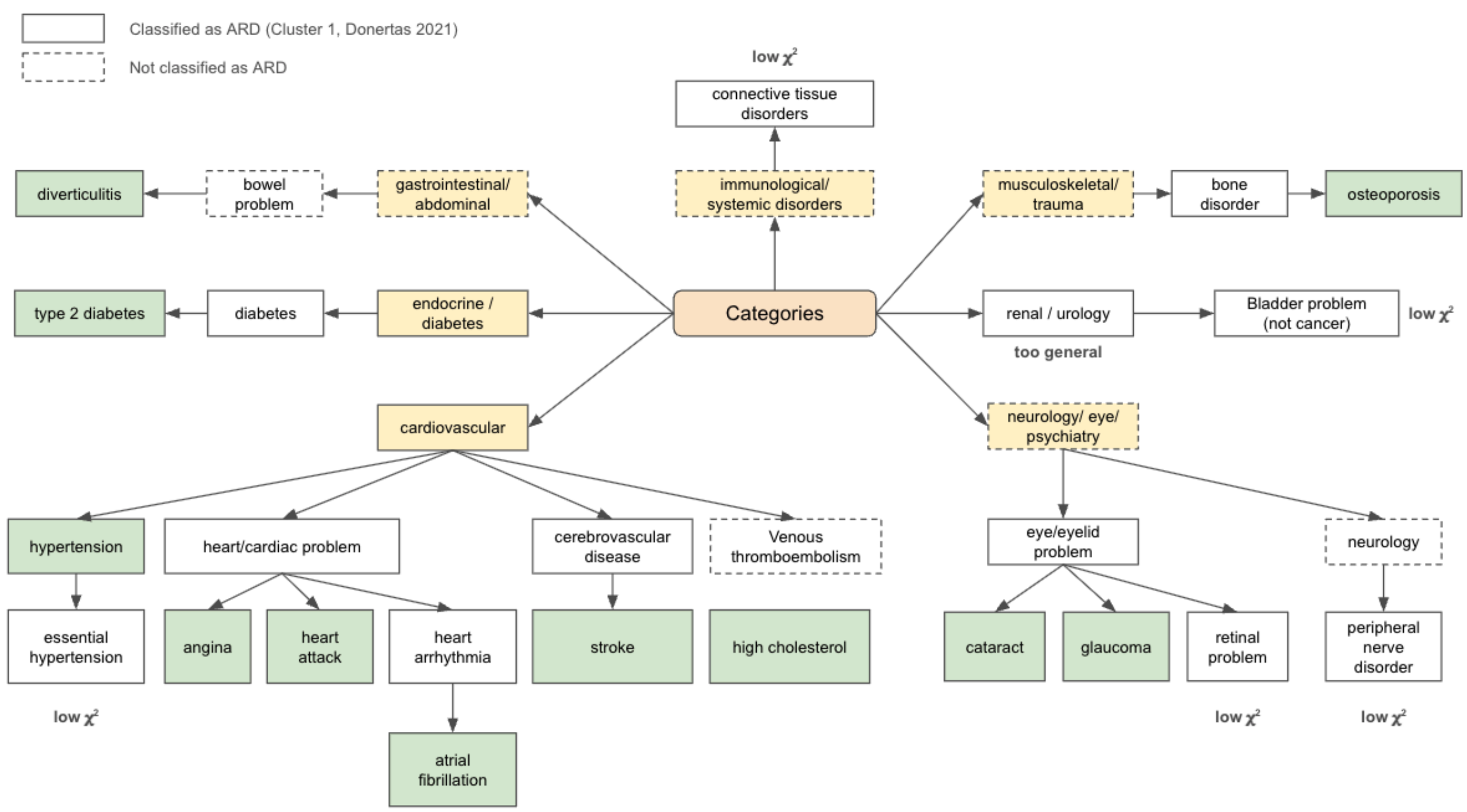

Supplementary Fig. S1 Hierarchical structure of age-related diseases (ARD). 25 common diseases whose age-of-onset profiles showed a sharp increase after age 40 were obtained from Donertas et al. (2021) and used to construct a hierarchical graph. Boxes highlighted in green indicate ARDs whose GWAS summary statistics met the inclusion criteria for our LDSC analysis

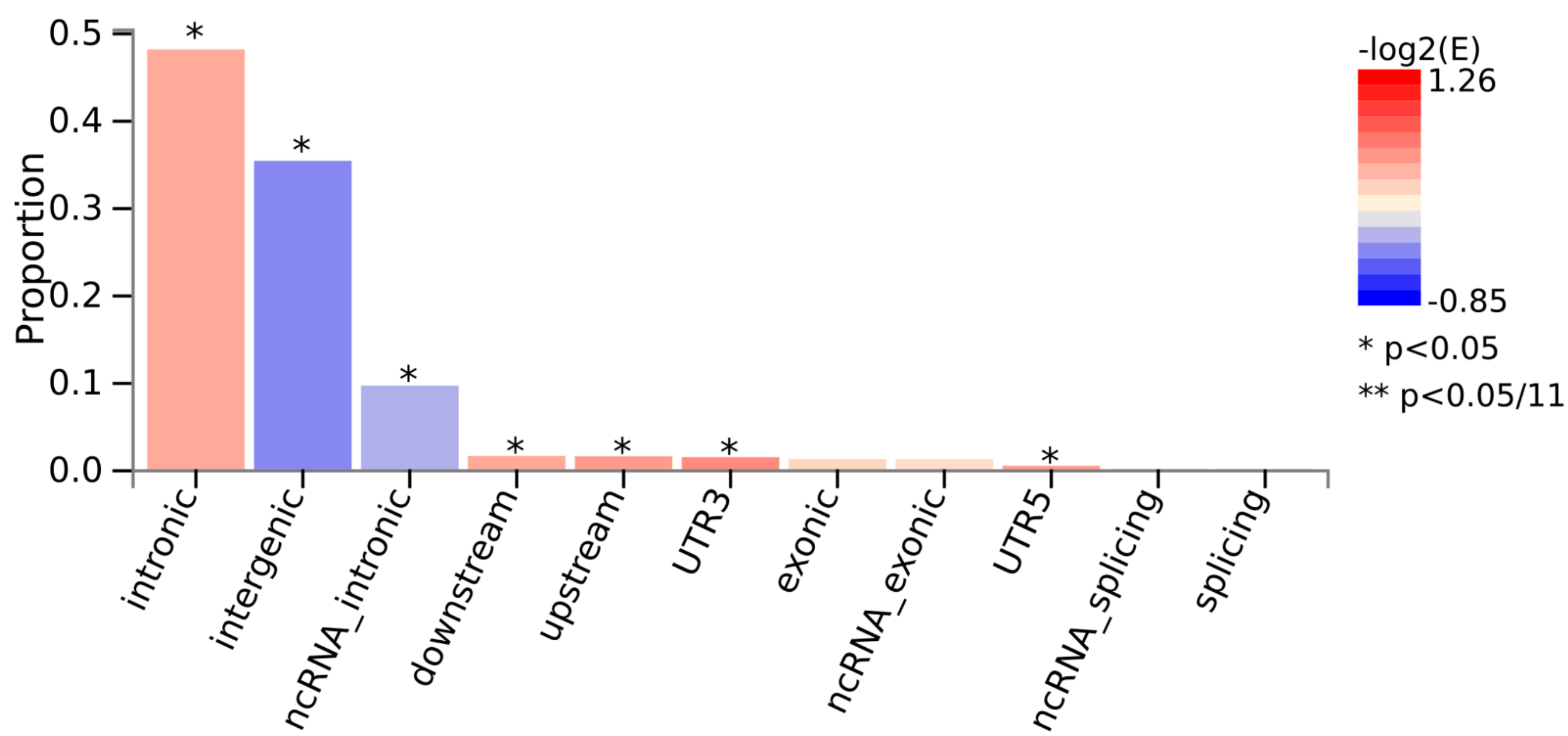

Supplementary Fig. S2 Functional consequences of SNPs on genes. The figure was generated by FUMA

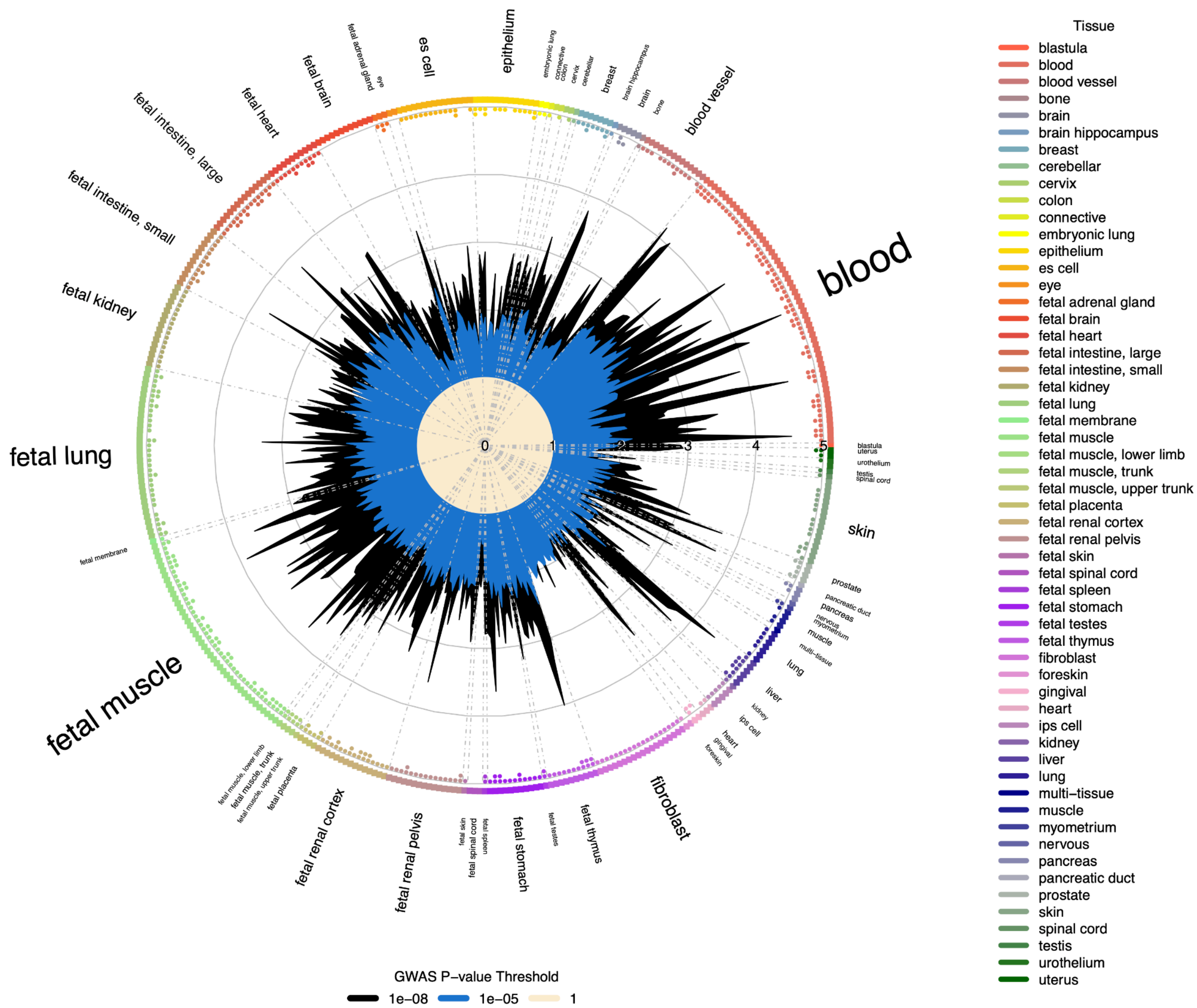

Supplementary Fig. S3 Enrichment of mvARD GWAS signals in open chromatin peaks across all ENCODE and Epigenomic DHS cell lines

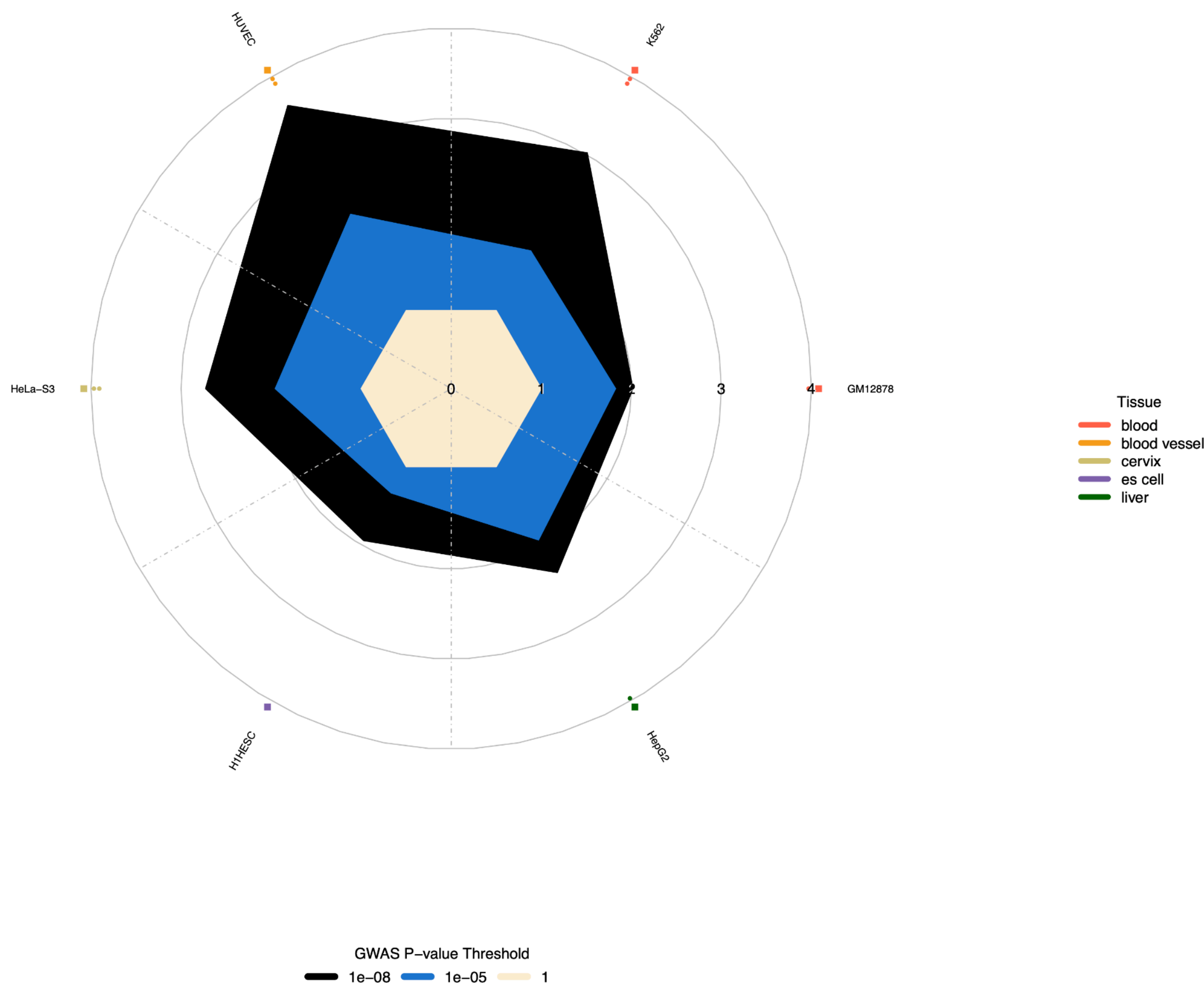

Supplementary Fig. S4 Enrichment of mvARD GWAS signals in formaldehyde-assisted isolation of regulatory elements (FAIRE)
